# Protein Nanocages as Building Blocks for Conjugated Supramolecular Materials Displaying Multitasking Properties

**DOI:** 10.1007/s12010-025-05364-4

**Published:** 2025-08-30

**Authors:** Hugo César Santillán-Uribe, Iris Ashanty Soto-Valerio, Juan Carlos León-Contreras, Ismael Bustos-Jaimes

**Affiliations:** 1 Departamento de Bioquímica, Facultad de Medicina, Universidad Nacional Autónoma de México, Mexico City, 04510 Mexico; 2 Laboratorio de Microscopia Electrónica, Departamento de Patología, Instituto Nacional de Ciencias Médicas y Nutrición Salvador Zubirán, Mexico City, 14080 Mexico

**Keywords:** Virus-like particles, Protein nanocages, Parvovirus B19, Bioconjugation, SpyTag-SpyCatcher

## Abstract

Protein nanocages are a group of compartments naturally enclosing nucleic acids or proteins for biological purposes. Such materials have also inspired the design of novel proteins displaying self-assembling properties. The most studied protein nanocages are viral capsids and their derivative virus-like particles (VLPs), which consist of any or all of the structural proteins of the virion but lack nucleic acids and are therefore non-infectious. VLPs can be used as vaccine antigens or decorated with heterologous antigens to develop new vaccine materials. External surfaces of VLPs can also be decorated with chemical substances to impart new properties, like fluorescence tags or binding to cellular receptors. In addition, the internal space of VLPs can be used to encapsulate therapeutic materials that can be carried to specific cells or tissues. Although VLPs are naturally polyvalent and can display more than one decorating element, it is possible to expand the repertoire of decorating species by specifically conjugating different VLPs, which can be decorated with different functional elements. Here, VLPs of parvovirus B19 displaying different functional proteins were linked through the SpyTag-SpyCatcher biorthogonal conjugation technology. Characterizing the resulting species demonstrated the bioconjugation and the presence of the individual properties of each component. This proof-of-concept research implies that novel multitasking biomaterials can be constructed from protein nanocages and that the application landscape is vast and highly expandable.

**Supplementary Information:**

The online version contains supplementary material available at 10.1007/s12010-025-05364-4.

## Introduction

The development of nanotechnology has allowed the construction of novel complex materials capable of performing highly specialized functions [1]. Many research groups worldwide invest considerable human and financial resources in developing this class of materials. Industries related to the preservation of health and the environment are not the exception [2]. Biomedical and nanomedical research is currently developing nanobiomaterials for delivering drugs or molecules of interest, devices that function as biosensors or imaging agents, and for generating vaccines and immunotherapies [3, 4]. Several examples of licensed VLP-based vaccines include those for Hepatitis B (Recombivax HB, Engerix-B, Eplisav-B, PreHevbrio), Hepatitis E (Hecolin), Human Papillomavirus (Gardasil, Gardasil 9, Cevatrix, Cecolin, Walrinvax), Malaria (Mosquirix), Japanese encephalitis virus (ChimeriVax-JE), SARS-CoV-2 (NVX-Cov2373, Covifenz), and there are others currently in clinical trials [5]. One of the most critical challenges for nanobiotechnology materials for therapeutic use is demonstrating their biocompatibility [6]. A first approach to achieving this characteristic can be raised through viruses, specifically virus-like particles (VLPs), since they have proven excellent platforms for obtaining functional and biocompatible materials. VLPs are self-assembling supramolecular arrangements of some of the structural elements in viruses (i.e., proteins) [7–9]. These particles preserve many properties of nanobiotechnological interest (e.g., dimensions on the nanometric scale, monodisperse distribution, and polyvalence). As proteinaceous materials, their components can be produced in heterologous systems to ease their manufacture [10]. For example, Primate Erythroparvovirus 1, usually parvovirus B19 (B19V), has been used to construct VLPs [11].

B19V is a pathogenic human virus that can cause various diseases in different age groups. B19V virions have dimensions ranging from 18 to 25 nm, and their genome consists of a ssDNA with a length of 5596 nt [12–14]. This genetic material is enclosed in an icosahedral capsid consisting of 60 protein monomers, of which approximately 95% of the protomers correspond to the VP2 protein and the remaining 5% to the VP1 protein. The difference between VP1 and VP2 is an additional trait of 227 amino acid residues at the N-terminus of VP1, the so-called VP1 unique region (VP1u) [15–18]. VP2, VP1, or both proteins can be used for the in vitro assembly of VLPs with different properties [19–25]. The in vitro assembly of B19V VLPs proceeds through the resuspension and further refolding of inclusion bodies produced by the expression of the recombinant viral protein. Moreover, chimeric forms of the protein VP2 have been constructed and used in VLP assembly. This type of VLP assembly allows the manufacture of hybrid particles composed of more than one chimeric protein whose components confer the combined properties to the resulting VLPs [26, 27]. The decoration of B19V VLPs with the bioconjugating peptide SpyTag has allowed the incorporation of heterologous functional proteins previously fused to the bioconjugation partner of SpyTag, the SpyCatcher domain. This system allows the covalent union of complementary fragments of a protein through an isopeptide bond [28]. This conjugation method is critical for proteins that cannot be fused to the coat protein VP2, as the refolding and assembly conditions can be harsh for many proteins [29].

The main goal of this research was to exploit the properties of these particles to produce new nanobiomaterials with a non-previously reported supramolecular architecture. VLPs have already been included in hydrogels to provide new properties in these materials used as bioactive scaffolds for tissue regeneration [30]. Also, higher-order assemblies of viral particles to generate 2D/3D microscale assemblies through non-covalent interactions have been reported [31]. In contrast with our approach, such assemblies were composed of homogeneous capsids, sometimes reaching the scale of centimeters. However, in our approach, keeping the diameter below 100 nm is important so as not to risk the nanobiomaterial’s solubility. Moreover, we aimed to demonstrate the gain of functions as a result of the binding of the nanoparticles. Therefore, the SpyCatcher and SpyTag sequences were genetically inserted into the external surface loop 301–313 of the structural protein VP2 of B19V, and the chimeric proteins were used to assemble VLPs. Additionally, the protein SpyCatcher-VP2 [26] was utilized to construct hybrid VLPs by co-refolding this protein with a VP2 chimera that incorporated the *Bacillus pumilus* lipase into the same surface loop [26, 32]. This loop was selected for the insertion of heterologous elements in B19V VLPs, as it displayed no electron density in the crystallographic structure (PDB 1S58), suggesting that it is not critical for the particle assembly [11, 33]. The produced VLPs were used to conjugate with each other. Finally, the remaining SpyTag peptides were conjugated with a chimera of the superfolder GFP (sfGFP) [34] with the SpyCatcher domain, as previously reported [27]. The resulting species were analyzed using hydrodynamic techniques, and their composition, fluorescence, and hydrolytic properties were analytically confirmed.

## Materials and Methods

### Chemicals and Biochemicals

All chemical reagents used were analytical grade and purchased from Sigma-Aldrich. The SDS-PAGE protein ladder was purchased from Life Technologies (Gibco BRL). Anti-B19V Antibody, aa 328–344 of VP2 capsid protein clone R92F6, and Polyclonal anti-mouse Ig-G (Fab-specific)-peroxidase antibody produced in goats was purchased from Merck. The chemiluminescent substrate used to detect horseradish peroxidase (HRP) was WesternSure Premium, LI-COR, Inc.

Genes were chemically synthesized (Epoch Life Science, Inc.) and independently cloned into the pET22b(+) vector (Invitrogen) between the NdeI and XhoI restriction sites in the same ORF of a 6xHis Tag at the C-termini provided by the vector to simplify the purification by IMAC in denaturing conditions. The sequences of all chimeric proteins are found in supporting information in Table S1.

### Expression and Purification of Proteins

We used *Escherichia coli* BL21(DE3) (Invitrogen, USA) to express all the recombinant proteins. The plasmids for all the proteins were used to transform *E. coli* BL21(DE3) competent cells by the CaCl_2_ and heat shock method. Transforming colonies were selected from LB agar plates supplemented with 100 µg/mL Ampicillin incubated overnight at 37 °C.

We inoculated 400 mL of LB-Amp medium (100 µg/mL) with cells containing the interest plasmids for protein expression. The medium was incubated at 37 °C and orbital shaking until reaching an OD_600_ = 0.6. Subsequently, we added isopropyl β-D-thiogalactopyranoside (IPTG) to the culture media as an inducing agent up to 0.25 mM. The culture medium containing the cells that expressed the GFP-SC protein was incubated for 6 h at 30 °C and orbital agitation at 220 rpm. The remaining culture media were incubated for 16 h at 30 °C and 220 rpm. After the periods described, the cells were harvested by centrifugation at 5000 rpm for 30 min at 4 °C. The biomass was washed twice with a 0.9% m/v NaCl solution and stored at − 20 °C. The cell pellet was resuspended in washing buffer (50 mM Tris–HCl, 150 mM NaCl, 5 mM EDTA, pH 8.0), and the cells were harvested by centrifugation at 5000 rpm for 15 min at 4 °C. The pellet was resuspended in lysis buffer (50 mM Tris–HCl, 150 mM NaCl, 5 mM EDTA, 2% v/v Tritón-X100, pH 8.0) and then lysed by sonication using 20 s pulses with 20 s off during 10 min in iced water bath. Inclusion bodies were collected by centrifugation at 8000 rpm for 20 min at 4 °C. The pellet was rewashed with lysis buffer by sonication under the same conditions, and the insoluble fraction containing inclusion bodies was pelleted by centrifugation at 10,000 rpm for 40 min at 4 °C. The pellet was solubilized in solubilization buffer (50 mM NaH_2_PO_4_, 0.3 M NaCl, 2 mM DTT, 5 M GdnHCl, pH 6.3) by incubation for 12 h at 30 °C with orbital shaking of 200 rpm. Purification of His-tagged proteins was performed under denaturing conditions in a 15-mL column of IMAC resin (Protino Ni-TED, Macherey Nagel) equilibrated with column buffer (50 mM NaH_2_PO_4_, 0.3 M NaCl, 3 M GdnHCl, pH 7.5). The column was washed with 50 mL of column buffer, then with 30 mL of column buffer added with 15 mM imidazole, and finally with 30 mL of column buffer added with 30 mM imidazole. The protein was eluted with 30 mL of elution buffer (50 mM NaH_2_PO_4_, 0.3 M NaCl, 3 M GdnHCl, 0.3 M Imidazole, pH 7.5) and collected in 5 mL fractions. Protein fractions were analyzed by SDS-PAGE, and those containing the pure proteins were pooled and concentrated with centrifugal filter units (Millipore) in a 30-kDa molecular weight cutoff membrane. During centrifugal filtration, the buffer was changed to storage buffer (20 mM Tris–HCl, 0.15 mM NaCl, 1 mM EDTA, 1 mM DTT, Gdn-HCl 5 M, pH 8). The protein concentration was estimated by the bicinchoninic acid assay (BCA) and 280 nm absorbance.

For the expression and purification of sfGFP-SC, transformed cells of *E. coli* BL21(DE3) were grown at 37 °C in LB-Amp medium (100 µg/mL) to an OD_600_ ~ 0.6 and protein expression was induced by the addition of IPTG to a final concentration of 0.5 mM; then incubation was continued for 12 h. Cells were harvested by centrifugation at 5000 rpm for 15 min and washed two times with phosphate buffer 50 mM, pH 7.5. The cells were collected by centrifugation at 5000 rpm for 10 min at 4 °C and then resuspended in the same buffer added with 50 mM imidazole and disrupted by sonication using 20 s pulses with 20 s off during 10 min in an iced water bath. The cell-free extract was centrifuged at 10,000 rpm for 20 min to remove cell debris. Ten milliliters of this crude extract was loaded into a column packed with 15 mL of IMAC matrix Protino Ni-TED equilibrated with loading buffer (20 mM KH_2_PO_4_, 0.5 M NaCl, 20 mM Imidazole, pH 7.5). The column was washed with 20 column volumes of loading buffer. Elution was achieved by washing the column with the elution buffer (20 mM KH_2_PO_4_, 0.5 M NaCl, 0.5 M imidazole, pH 7.5). The proteins were dialyzed against PBS buffer pH 7.4, except for sfGFP-SC, which was dialyzed against PBS pH 7.4 added with 50% glycerol.

### Refolding and Self-Assembly of VLPs

VLP assembly was carried out by dialysis of 1.5 mL of the solubilized recombinant protein (0.7 mg/mL in solubilization buffer) against 50 mL of PBS-2X-Arg (274 mM NaCl, 5.4 mM KCl, 20 mM Na_2_HPO_4_, 4 mM KH_2_PO_4_, 0.25 M L-Arg, pH 7.4) supplemented with 0.2 M of L-Arg, at 4 °C, changing buffer every 12 h over a 48-h period [19]. The proteins were used in a 1:1 ratio for the assembly of hybrid particles, and the final concentration of protein was kept at 0.7 mg/mL.

Size exclusion chromatography (SEC) was applied as a preparative purification method to remove proteins by their hydrodynamic diameters. The eluted peaks containing SpyTag-VP2 protein were passed through a size exclusion column Sephacryl S-500 HR (Cytiva) packed column, equilibrated in PBS-Arg buffer (137 mM NaCl, 2.7 mM KCl, 10 mM Na_2_HPO_4_, 2 mM KH_2_PO_4_, 0.2 M L-Arg, pH 7.4) with a flow of 0.5 mL/min, using an FPLC system (Äkta Purifier, GE Healthcare). The samples collected were analyzed by DLS to confirm the presence of VLPs or the corresponding clusters.

### SpyTag-SpyCatcher Bioconjugation

The bioorthogonal conjugation reaction was performed by mixing the SpyCatcher/SpyTag in a molar ratio of 2:1 in the first conjugation stage and 1:1 in the second stage. The decrease in the SpyCatcher/SpyTag ratio during the second stage is because of the high yield of the reaction between its components. The reaction was carried out in PBS 2X-Arg and incubated in a Thermomixer (Eppendorf) at 10 °C for 48 h. These conditions were selected after testing various temperatures and incubation times to achieve high yield and minimal protein degradation. The samples were passed through a Sephacryl S-500 HR column to separate the unconjugated proteins. The samples were analyzed by SDS-PAGE and DLS to confirm the bioconjugation.

### DLS Analysis

VLP particle size was determined by dynamic light scattering (DLS) in a Zetasizer μV (Malvern) using a dispersant refractive index of 1.33 as measured in an Abbe refractometer NAR-3 T (Atago), and the viscosity was assumed to be 1.003 cP [35]. All samples were purified by SEC in a Sephacryl S-500 HR column as described earlier, and then filtered through 0.45 and 0.22 μm polyvinylidenefluoride (PVDF) syringe filters (Millipore), and protein concentration was adjusted between 0.2 and 0.5 mg/mL. Measurements for each sample were averaged over 5 runs of 10 measurements per run at 10 °C.

### TEM Analysis

The samples eluted from the IMAC column were diluted 1:50 in PBS, filtered through a 0.22 µm membrane filter, fixed for 5 min on Formvar-coated copper grids, and negatively stained with 1% (w/v) phosphotungstic acid (PTA), pH 7.0, for 5 min. Samples were imaged using a Tecnai T12 TEM (Philips) at 80 kV.

### Western Blot Analysis

Semi-wet Western blot for VP2 detection in assemblies of chimeric proteins.

An SDS-PAGE was made with two sets of samples to transfer into the PVDF membrane. Before the transfer protocol, the membrane and SDS-PAGE were saturated with transfer buffer (0.84 g NaHCO_3_, 0.32 g Na_2_CO_3_, 200 mL methanol, sterile distilled H_2_O to 1 L) for 30 and 15 min, respectively. The transfer protocol was performed at 15 V for 30 min. Western blot was performed for the antibody anti-VP2. First, the PVDF membrane was blocked for 1 h with 5% milk in TBS-Tween (2.42 g Tris-base, 29.2 g NaCl, 1 mL Tween-20, sterile distilled H_2_O to 1 L). After that, the PVDF membrane was rinsed with TBS-Tween and washed three times with TBS-Tween for 10 min. Then, the primary antibody, an anti-B19V antibody (1:7000 dilution), was incubated for 1 h. Immediately, three washes with TBS-Tween for 10 min were performed. The second antibody was incubated with HRP at 1:4000 for 1 h. After three washes with TBS-Tween for 10 min, the presence of the secondary antibody was revealed with a mix of 500 µL of the luminol solution with 500 µL of buffer for 30 s. Immediately, the PVDF membrane was placed into the Licor C-Digit equipment. All the washes were performed with constant agitation.

### Electrophoretic Mobility Shift Assay

For the electrophoretic mobility shift assay (EMSA), a two-layer gel was set up, with the lower layer made of 1% agarose that supported the separation gel in the upper layer. The separation gel was 0.4% agarose. Agarose was prepared in GAE buffer (40 mM Glycine, 20 mM Acetic Acid, 1 mM EDTA, pH 9.0), and the same buffer was used for the electrophoresis chamber. Protein samples were concentrated to 25 µL, mixed with 15 µL of gel loading buffer (20% Glycerol, 0.2% Bromophenol Blue, 120 mM Tris Base), and loaded into the gel. The gel was run for 12 h at 20 V. The gel was stained with SYPRO Ruby (ThermoFisher) following the manufacturer’s instructions.

### Esterase Activity Assay

Esterase activity was measured by mixing 100 µL of protein sample with 850 µL of PBS pH 7.4. The reaction was triggered by adding 50 µL of 10-mM 4-nitrophenyl acetate (4NPA) dissolved in acetonitrile. Color development was followed at 410 nm for 3 min in a spectrophotometer at 25 °C. The spontaneous hydrolysis of the substrate was considered to correct product development. A standard curve of 4-nitrophenol in PBS at pH 7.4 was constructed to measure the amount of product.

## Results and Discussion

**Fig. 1:**
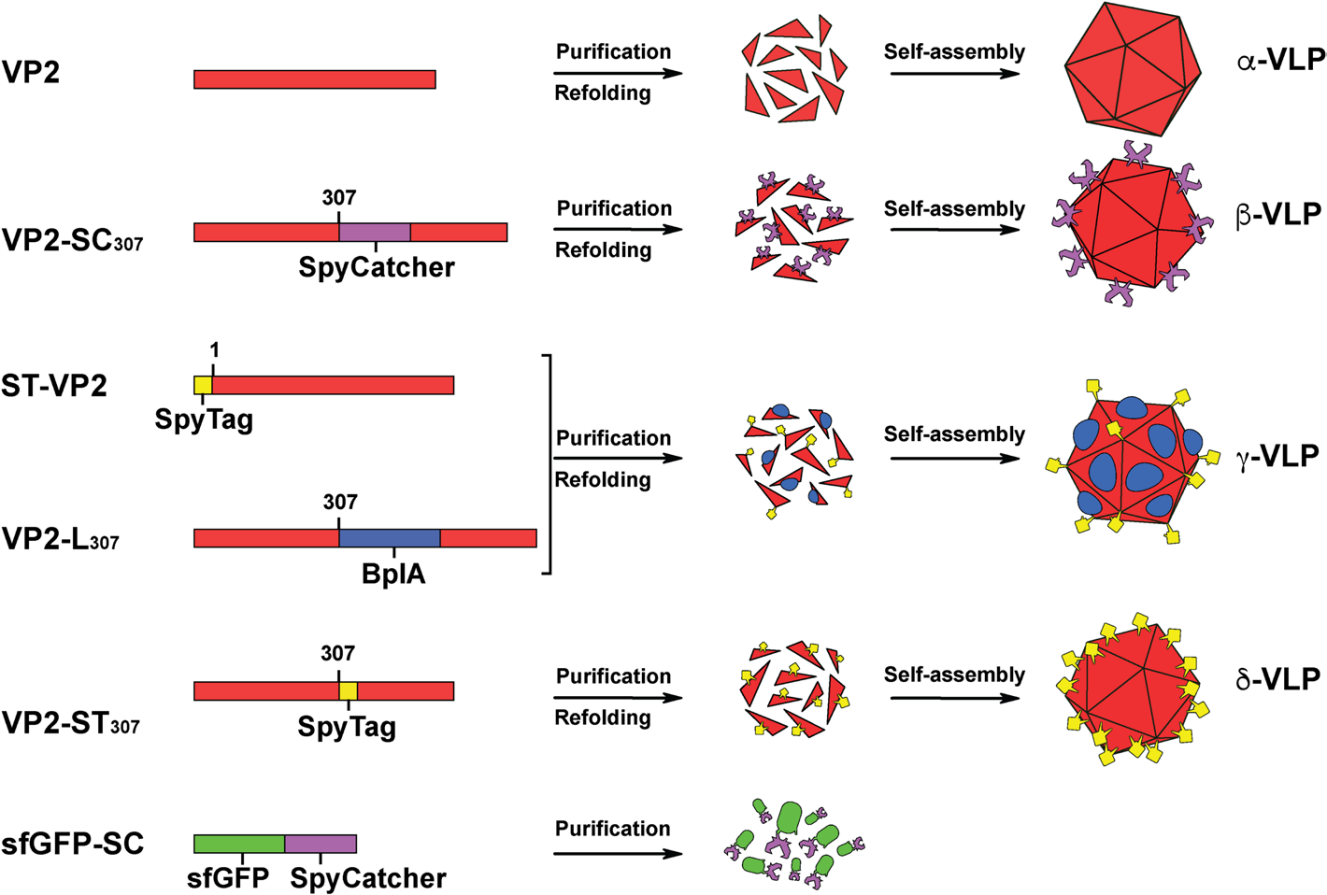
Properties and association forms of the recombinant proteins designed for constructing supramolecular materials used in this research. VP2 is the 6x-His tagged form of the wild-type B19V VP2; VP2-SC_307_ is VP2 harboring the SpyCatcher domain between residues 307 and 308 of VP2; ST-VP2 is VP2 with the SpyTag peptide fused at its N-terminal; VP2-L_307_ is VP2 harboring the *Bacillus pumilus* lipase domain between residues 307 and 308 of VP2; VP2-ST_307_ is VP2 harboring the SpyTag peptide between residues 307 and 308 of VP2; sfGFP-SC is the superfolder GFP fused to the SpyCatcher domain

This research involved six recombinant proteins (Fig. 1). VP2 is the previously described protein for VLP assembly [35]. VP2-SC_307_ is a form of VP2 with the SpyCatcher domain inserted between residues 307 and 308. ST-VP2 is VP2 fused to the SpyTag peptide at its N-terminus and has been reported before [27]. VP2-L_307_ is a form of VP2 with a lipase inserted between residues 307 and 308, and it was also previously studied [26]. VP2-ST_307_ is a form of VP2 with the SpyTag peptide inserted between residues 307 and 308. Finally, the last protein used here is the superfolder GFP (sfGFP) fused to the N-terminus of the SpyCatcher domain, sfGFP-SC, reported elsewhere [27].

VP2 self-assembles into VLPs, which are designated here as α-VLPs and are used as a control. VP2-SC_307_ was designed to display the SpyCatcher domain on the external surface of the VLPs, which were designated as β-VLP. ST-VP2 and VP2-L_307_ have individually been demonstrated to self-assemble into VLPs. In this study, these proteins are expected to form a hybrid particle displaying both heterologous elements, SpyTag and the lipase, on the external surface of assembled particles called -VLPs. The chimeric protein VP2-ST_307_ is intended to self-assemble into VLPs displaying the SpyTag peptide on their external surface, and these were designated δ-VLPs. The last protein, sfGFP-SC, has to participate as an imaging probe for molecular tracking and as a terminator by blocking any SpyTag peptide excess.

**Fig. 2:**
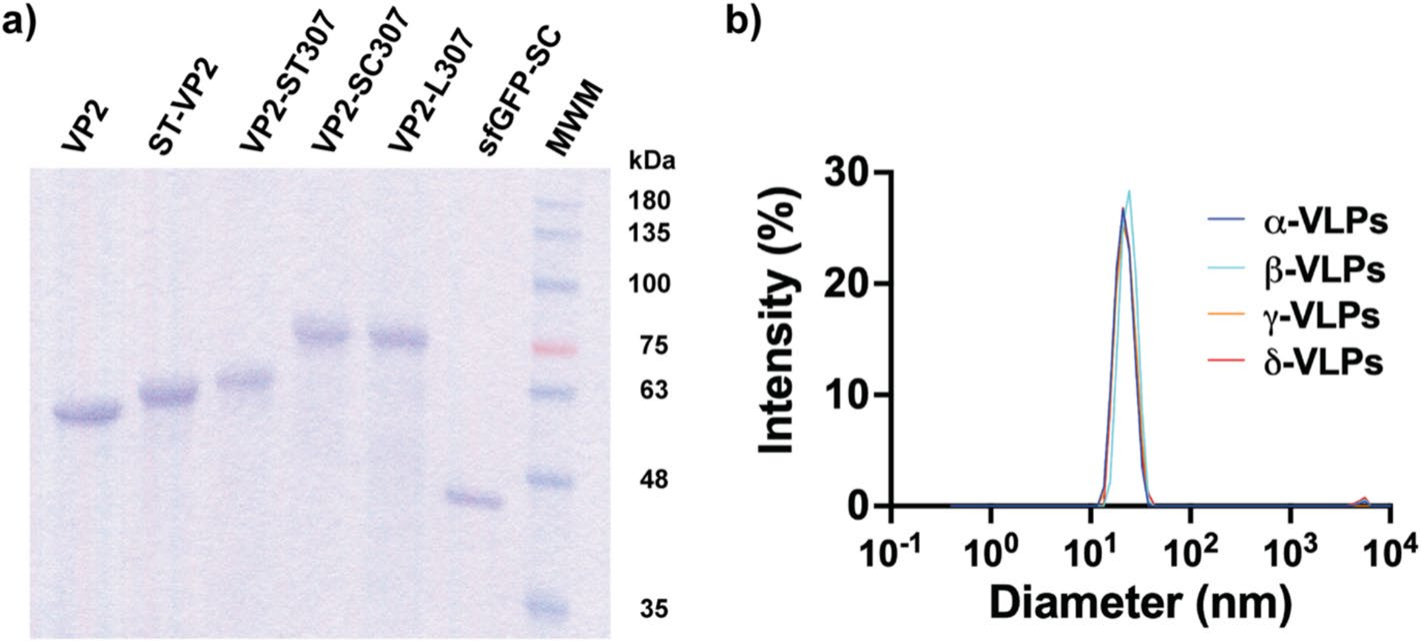
Analysis of the purified proteins and evaluation of their self-assembly ability. SDS-PAGE analysis of the purified proteins: VP2, 61 kDa; ST-VP2, 64 kDa; VP2-ST_307_, 65 kDa; VP2-SC_307_, 76 kDa; VP2-L_307_, 81 kDa; sfGFP-SC, 44 kDa (**a**). DLS analysis of the assembled VP2 chimeras with diameters of 21.8 ± 4.3 nm for the α particles, 22.5 ± 4.7 nm for the β particles, 24.0 ± 4.4 nm for the γ particles, and 22.4 ± 4.6 nm for the δ particles (**b**)

The six proteins were expressed and purified to electrophoretically pure species (Fig. 2a). To assess the ability of all the VP2 forms to self-assemble into VLPs, the proteins underwent the refolding/self-assembly protocol followed by SEC purification. Then, they were analyzed by DLS to estimate their diameter (Fig. 2b).

**Fig. 3:**
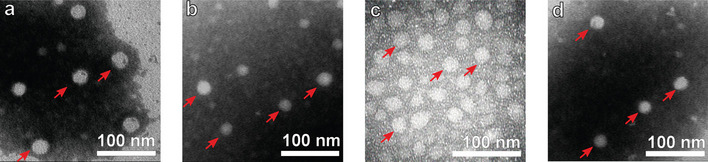
Transmission electron microscopy of the α (**a**), β (**b**), γ (**c**), and δ (**d**) particles. Samples were contrasted with PTA

The size distribution profiles showed that the β, γ, and δ particles have a hydrodynamic size remarkably similar to the α particles that we use as a control for the formation of VLPs (Fig. 2b). The α particles are made up of VP2 protomers and have a diameter of 21.8 ± 4.3 nm. The β particles comprised of the VP2-SC_307_ protomers have a 22.5 ± 4.7 nm diameter. On the other hand, hybrid γ particles, made up of a combination of the ST-VP2 and VP2-L_307_ protomers in a 1:1 molar ratio, showed a diameter of 24.0 ± 4.4 nm. Lastly, the δ particles made up of VP2-ST_307_ protein had a 22.4 ± 4.6 nm diameter. Those sizes are in agreement with the previous results for VP2, ST-VP2, and VP2-L_307_ VLPs [26, 27, 29]. In addition, the hybrid γ particles showed esterase activity against 4-nitrophenyl acetate (4NPA) (Table 1). Remarkably, the non-previously studied proteins carrying the SpyCatcher and SpyTag insertions at position 307 appear to self-assemble into VLPs. Finally, the α, β, γ, and δ particles were analyzed by TEM, confirming the typical morphology and size of parvovirus-like particles (Fig. 3).

**Table 1:** Esterase activity of the species containing the lipase domain (VP2-L_307_)

Species	Specific activity (U/mmol)*
γ-VLPs	11.7 ± 0.4
β-γ cluster	10.2 ± 0.6
β-γ-δ cluster	8.6 ± 1.3

*μmol of product min^−1^ μmol^−1^ lipase domain

**Fig. 4:**
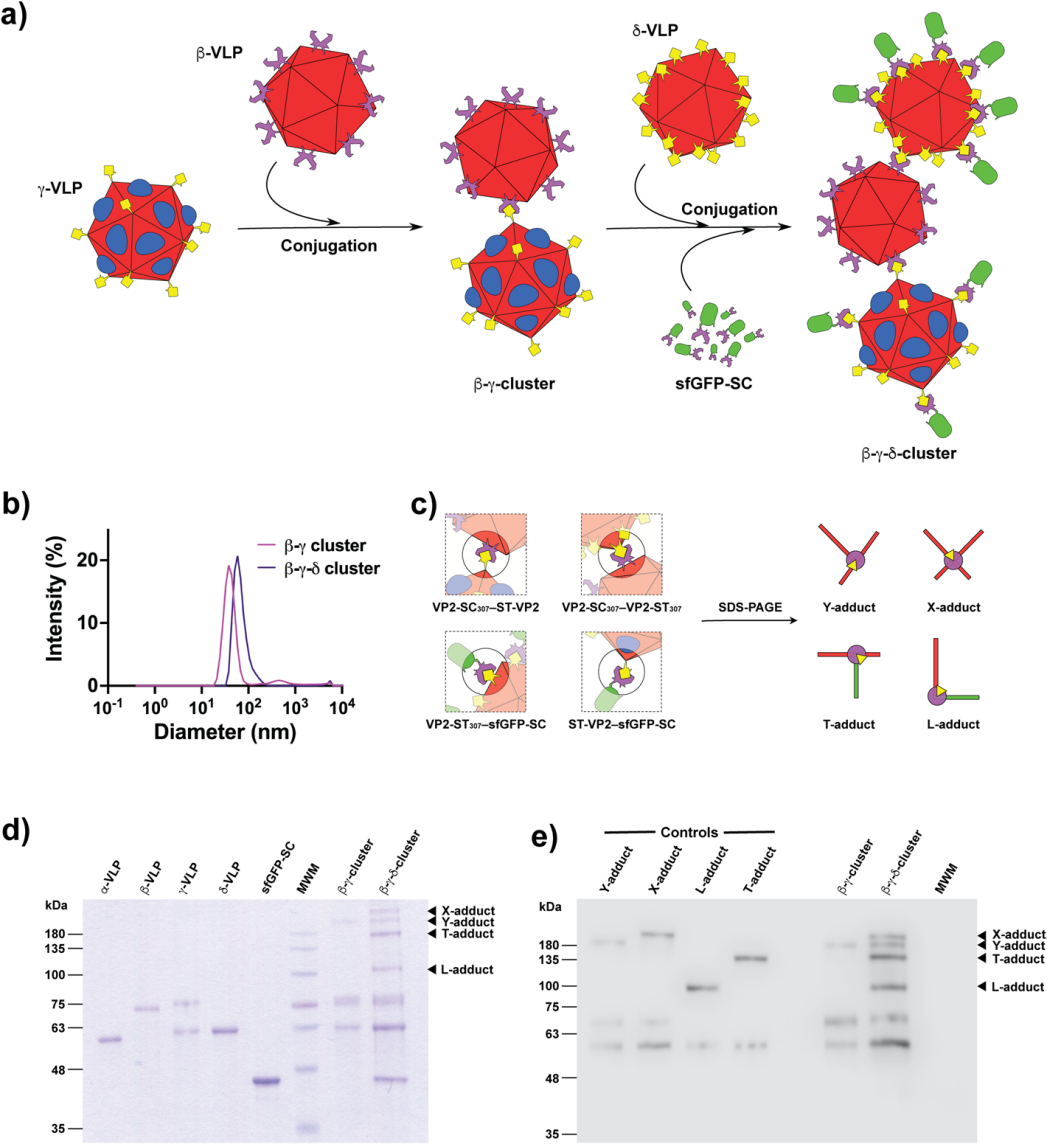
Construction of β-γ and β-γ-δ clusters from the building block VLPs. Schematic representation of the construction of the clusters (**a**). DLS analysis of the β-γ and β-γ-δ clusters with average diameters of 40.9 ± 12.2 and 69.8 ± 27.7 nm, respectively (**b**). All the possible conjugation modes between the chimeric proteins and a schematic view of their branching patterns (**c**). SDS-PAGE analysis of the building block proteins and the purified β- and β--δ clusters (**d**). Western blot of the control interactions shown in panel c and the purified β-γ and β-γ-δ clusters using an antibody against protein residues 328–344 of VP2, which is present in all the VP2 chimeras (**e**)

The second step in the building of new materials using VLPs as building blocks was to conjugate suitable partners. The β particles have the SpyCatcher component on the surface and are, therefore, capable of coupling with γ and particles that display the SpyTag peptide on their surface. In the first coupling stage, the β and γ particles were incubated to obtain the β-γ cluster (Fig. 4a). The DLS analysis of the resultant species revealed a diameter of 40.9 ± 12.2 nm (Fig. 4b). In comparison, the parental species had diameters of ~ 23 nm. Surprisingly, only a tiny fraction of larger-sized species was detected. Considering that the SpyTag peptide is present in the γ particles in a 1:1 ratio and that the particle has 60 subunits, the particles should be composed of ~ 30 subunits of ST-VP2. Contrastingly, there are ~ 30 VP2-L_307_ protomers, displaying the lipase domain on the external loop 301–313 of the VP2 protein. There are two plausible reasons for these particles not to grow indefinitely, forming a 3-D grid and developing a gel-like texture. The first is that previous research has shown that not all the N-terminus regions of chimeric VP2 subunits are externalized through the fivefold symmetry pore. Previous results with a different peptide showed that only ~ 35% of the chimeric N-termini were exposed on the external surface of B19V VLPs [36]. Thus, the number of SpyTag peptides could be significantly lower than 30 (~ 10). The second reason is the steric hindrance provoked by the ~ 30 lipase domains and the first β-VLP, making it difficult for other β-VLPs to get close to conjugate with the few remaining free SpyTag peptides. Consider that the SpyTag is 25 residues long (including the linker) and is present in a much smaller number (< < 30), while the lipase domain is a 183-residue globular domain. The β-γ cluster is shown in Fig. 4a as two conjugated particles. However, this cluster could be a mixture of species composed of two or three conjugated particles, with the β particle as an anchor structure. Such a structure would also be compatible with the size range detected by DLS. However, for the conjugation of the δ VLPs, it is essential to have some free SpyCatcher domains in the β VLPs.

To confirm the covalent interaction between β- and γ-VLPs, and the presence of the non-conjugated VP2-SC_307_ subunits of the γ particles, the resulting species were analyzed by SDS-PAGE (Fig. 4d). The β-γ cluster clearly shows all of its parental components and a non-previously present band of high molecular mass, > 180 kDa. The sum of the molecular masses of ST-VP2 and VP2-SC_307_ is ~ 140 kDa; thus, the mass of this new adduct disagreed with the theoretical mass of the VP2-SC_307_–ST-VP2 adduct. Nevertheless, considering that the conjugating elements are located in different sites along the linear sequence of VP2, it is possible to distinguish four different conjugation modes for the achievable adducts in our work scheme: VP2-SC_307_–ST-VP2, VP2-SC_307_–VP2-ST_307_, VP2-ST_307_–sfGFP-SC, and ST-VP2–sfGFP-SC. Such species can be classified according to the length of their branches into Y, X, T, and L adducts (Fig. 4c). Although the molecular masses of the Y and X (140 and 141 kDa), or T and L (109 and 108 kDa) adducts are alike, it is easy to rationalize that their mobility in SDS-PAGE will be different in agreement with its branched structure in denaturing conditions. As hypothesized, controls for the Y, X, T, and L adducts migrated differently (Fig. 4e), producing the Y-adduct with an apparent molecular mass > 180 kDa. It is worth noting that the L adduct migrates almost as a linear protein as the crosslinking point is at the end of the conjugating proteins. Thus, the L shape is only conceptual and is not related to the actual shape of the adduct.

For the second coupling stage of conjugation, the β-γ cluster was incubated with δ particles to induce the formation of a trimodular arrangement called the β-γ-δ cluster. The particles, in contrast to γ particles, have a SpyTag peptide on each subunit of the 60 subunits composing the VLPs, producing thus a very high density of conjugating elements on the surface of the particles. In such conditions, some δ particles were expected to have a good chance to conjugate with the anchor particle β. Finally, to stop the conjugation reaction and simultaneously label the β-γ-δ cluster, monomers of the GFP-SC protein were incorporated to complete the novel bionanomaterial. The produced species presented an average hydrodynamic diameter of 69.8 ± 27.7 nm by DLS (Fig. 4b), larger than the β-γ cluster by ~ 20 nm. Again, the new interactions between δ and β particles, and between sfGFP-SC and ST-VP2 or VP2-ST_307_, will produce adducts with particular branched patterns (Fig. 4c) that were successfully detected by SDS-PAGE and Western Blot (Fig. 4d, e). Note that the X-adduct with the most branched character shows the highest apparent molecular mass due to its anomalous migration in SDS-PAGE.

The β-γ-δ cluster showed esterase activity against 4NPA (Table 1). The loss in specific activity observed in the clusters is limited, but it could be the result of the time and manipulation of the sample because, although the lipase domain fused to the VP2 protein is more stable than the free enzyme, the thermal inactivation of the enzyme still occurs [26].

**Fig. 5:**
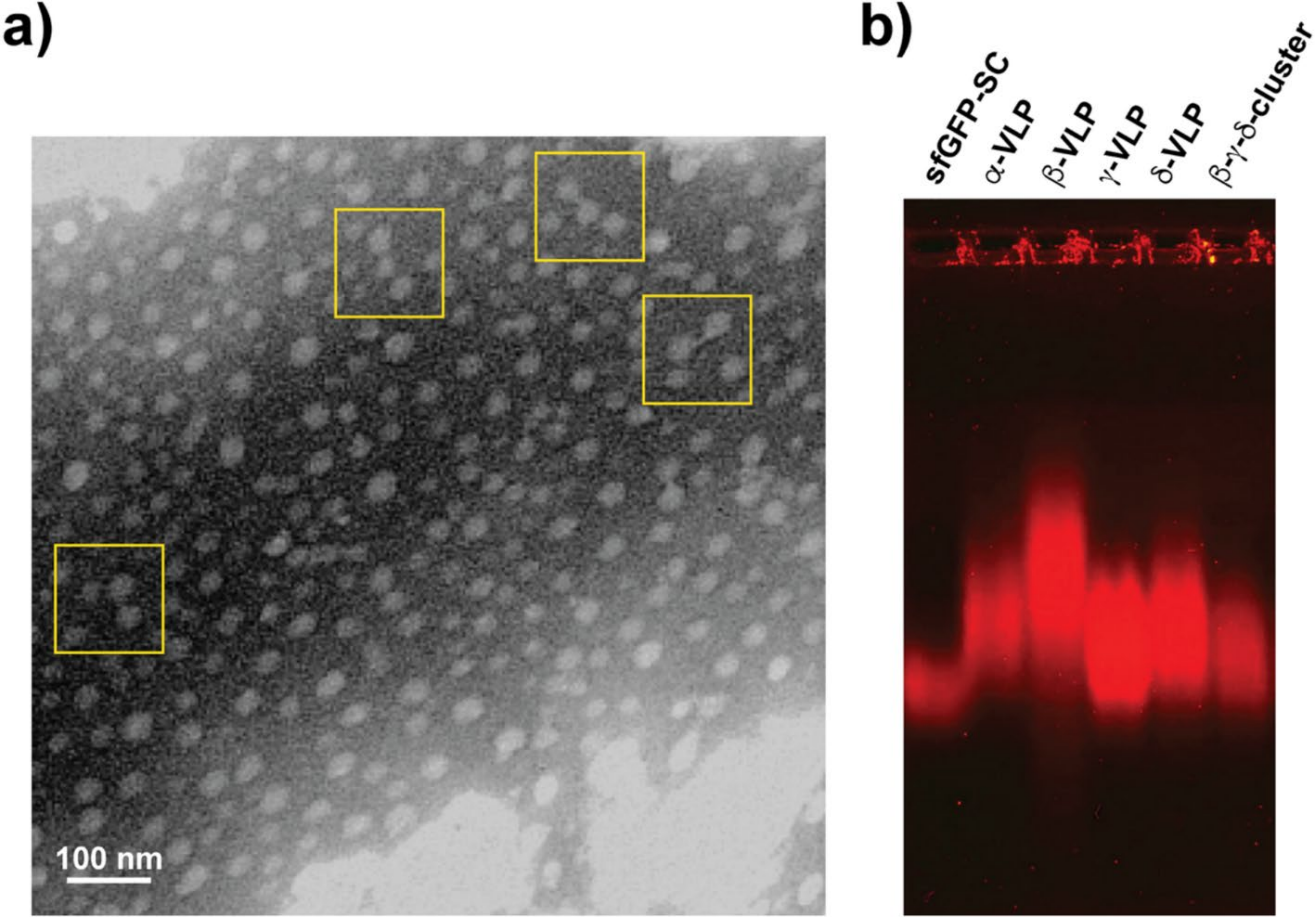
Analysis of the β-γ-δ cluster. Transmission electron microscopy (TEM) of the β-γ-δ cluster contrasted with PTA (**a**). Electrophoretic movement shift assay (EMSA) in 0.4% agarose of the β-γ-δ cluster and its building blocks stained with SYPRO Ruby (**b**)

The β-γ-δ cluster was also analyzed by TEM to observe their possible configurations (Fig. 5a). TEM images, however, show particles that appear to be independent, except for some of them that appear to be linked. It is possible that the locations where the interactions between particles were formed are not always in an adequate position to be imaged. Nevertheless, the presence of a few clearly linked particles implies that the bioconjugation occurred, as already detected by SDS-PAGE and DLS analyses.

The electrophoretic mobility shift assay (EMSA) in agarose gel was used to confirm the differences among the building blocks and the β-γ-δ clusters (Fig. 5b). Agarose gels have large pore diameters of about 1.5 µm for 0.4% [37], so this assay does not have enough resolution to resolve supramolecular species in the range of 20 to 100 nm as VLPs and the β-γ- clusters unless they show a significant difference in electric charge. Theoretical isoelectric pH (pI) for the proteins participating as building blocks is close, between 5.63 and 7.22; therefore, no very large changes in mobility were detected. However, taking together the small contributions of size and charge, detectable changes in electrophoretic mobility among species may be produced.

Ordering the proteins in increasing order of pI we have sfGFP-SC (5.63) < VP2-SC_307_ (6.02) < VP2 (6.53) < ST-VP2 (6.63) < VP2-ST_307_ (6.66) < VP2-L_307_ (7.22). The highest mobility in the assay was for sfGFP-SC, in agreement with its pI. However, the mobilities of the multisubunit particles did not follow the same behavior, perhaps because the calculated pI does not consider the oligomeric nature of the proteins forming VLPs. The mobility of the cluster β--δ was similar to the mobility of particles and showed no evidence of free β- or δ-VLPs or free sfGFP-SC. This result, as well as the measured esterase activity and observable fluorescence, confirmed this novel material’s conjugative nature and multifunctional character.

## Conclusion

Chimeras of the structural protein VP2 of B19V can be self-assembled in vitro to produce homomeric or heteromeric VLPs carrying different structural elements with definite functions. In this proof-of-concept research, it was demonstrated that the selection of heterologous elements to be added to these VLPs not only conferred catalytic or tagging properties to the resulting nanoparticles but also conferred the ability to build novel kinds of particles with a higher level of complexity and an expanded repertoire of possibilities for carrying functions. The β-γ-δ cluster manufactured here represents a modular platform that can perform three functions: enzymatic catalysis, the coupling between VLPs, and the binding of an imaging agent. However, it can be envisaged that the manufacturing of materials with combined catalytic, tagging, and imaging properties will meet the needs of new synergistic biologicals aimed at treating complex diseases such as cancer, diabetes, or autoimmune disorders. The specific and controlled synergy of all these structural and functional elements has great potential in medicine and biotechnology.

## Supplementary Information

Below is the link to the electronic supplementary material.

MOESM 1 (DOCX 14.5 KB)

## Data Availability

All data obtained or analyzed during this study are included in this article.
