# Supplementary material for: Protein Nanocages as Building Blocks for Conjugated Supramolecular Materials Displaying Multitasking Properties
Source: Appl Biochem Biotechnol. 2025 Aug 30;197(10):6918–31. doi: 10.1007/s12010-025-05364-4 (PMC12619782; doi:10.1007/s12010-025-05364-4)
Supplement: Supplementary file 1 — (DOCX 14.5 KB) [file 12010_2025_5364_MOESM1_ESM.docx]

**Supplementary material**

**Protein nanocages as building blocks for conjugated supramolecular materials displaying multitasking properties**

Hugo César Santillán-Uribe^1^, Iris Ashanty Soto-Valerio^1^, Juan Carlos León-Contreras^2^, Ismael Bustos-Jaimes^1^*

^1^ Departamento de Bioquímica, Facultad de Medicina, Universidad Nacional Autónoma de México, Mexico City 04510, Mexico.

^2^ Laboratorio de Microscopia Electrónica, Departamento de Patología, Instituto Nacional de Ciencias Médicas y Nutrición Salvador Zubirán, Mexico City 14080, Mexico.

* Corresponding author. Tel.: +52 55 5623 2260; e-mail address: ismaelb@unam.mx (I. Bustos-Jaimes).

**Table S1.** Protein sequences.

| **> VP2**  MTSVNSAEASTGAGGGGSNPVKSMWSEGATFSANSVTCTFSRQFLIPYDPEHHYKVFSPAASSCHNASGKEAKVCTISPIMGYSTPWRYLDFNALNLFFSPLEFQHLIENYGSIAPDALTVTISEIAVKDVTDKTGGGVQVTDSTTGRLCMLVDHEYKYPYVLGQGQDTLAPELPIWVYFPPQYAYLTVGDVNTQGISGDSKKLASEESAFYVLEHSSFQLLGTGGTATMSYKFPPVPPENLEGCSQHFYEMYNPLYGSRLGVPDTLGGDPKFRSLTHEDHAIQPQNFMPGPLVNSVSTKEGDSSSTGAGKALTGLSTGTSQNTRISLRPGPVSQPYHHWDTDKYVTGINAISHGQTTYGNAEDKEYQQGVGRFPNEKEQLKRLQGLNMHTYFPNKGTQQYTDQIERPLMVGSVWNRRALHYESQLWSKIPNLDDSFKTQFAALGGWGLHQPPPQIFLKILPQSGPIGGIKSMGITTLVQYAVGIMTVTMTFKLGLRKATGRWNPQPGVYPPHAAGHLPYVLYDPTATDAKQHHRHGFEKPEELWTAKSRVHPLEHHHHHH |
| --- |
| **> ST-VP2**  MAHIVMVDAYKPTKGGSGGSGGSGGMTSVNSAEASTGAGGGGSNPVKSMWSEGATFSANSVTCTFSRQFLIPYDPEHHYKVFSPAASSCHNASGKEAKVCTISPIMGYSTPWRYLDFNALNLFFSPLEFQHLIENYGSIAPDALTVTISEIAVKDVTDKTGGGVQVTDSTTGRLCMLVDHEYKYPYVLGQGQDTLAPELPIWVYFPPQYAYLTVGDVNTQGISGDSKKLASEESAFYVLEHSSFQLLGTGGTATMSYKFPPVPPENLEGCSQHFYEMYNPLYGSRLGVPDTLGGDPKFRSLTHEDHAIQPQNFMPGPLVNSVSTKEGDSSSTGAGKALTGLSTGTSQNTRISLRPGPVSQPYHHWDTDKYVTGINAISHGQTTYGNAEDKEYQQGVGRFPNEKEQLKRLQGLNMHTYFPNKGTQQYTDQIERPLMVGSVWNRRALHYESQLWSKIPNLDDSFKTQFAALGGWGLHQPPPQIFLKILPQSGPIGGIKSMGITTLVQYAVGIMTVTMTFKLGLRKATGRWNPQPGVYPPHAAGHLPYVLYDPTATDAKQHHRHGFEKPEELWTAKSRVHPLEHHHHHH |
| **> VP2-SC_307_**  MTSVNSAEASTGAGGGGSNPVKSMWSEGATFSANSVTCTFSRQFLIPYDPEHHYKVFSPAASSCHNASGKEAKVCTISPIMGYSTPWRYLDFNALNLFFSPLEFQHLIENYGSIAPDALTVTISEIAVKDVTDKTGGGVQVTDSTTGRLCMLVDHEYKYPYVLGQGQDTLAPELPIWVYFPPQYAYLTVGDVNTQGISGDSKKLASEESAFYVLEHSSFQLLGTGGTATMSYKFPPVPPENLEGCSQHFYEMYNPLYGSRLGVPDTLGGDPKFRSLTHEDHAIQPQNFMPGPLVNSVSTKEGDSSSTGGSGGAMVDTLSGLSSEQGQSGDMTIEEDSATHIKFSKRDEDGKELAGATMELRDSSGKTISTWISDGQVKDFYLYPGKYTFVETAAPDGYEVATAITFTVNEQGQVTVNGKATKGDAHIDGGSGGENLYFQSGGTGGGAGKALTGLSTGTSQNTRISLRPGPVSQPYHHWDTDKYVTGINAISHGQTTYGNAEDKEYQQGVGRFPNEKEQLKRLQGLNMHTYFPNKGTQQYTDQIERPLMVGSVWNRRALHYESQLWSKIPNLDDSFKTQFAALGGWGLHQPPPQIFLKILPQSGPIGGIKSMGITTLVQYAVGIMTVTMTFKLGLRKATGRWNPQPGVYPPHAAGHLPYVLYDPTATDAKQHHRHGFEKPEELWTAKSRVHPLHHHHHHHH |
| **> VP2-ST_307_**  MTSVNSAEASTGAGGGGSNPVKSMWSEGATFSANSVTCTFSRQFLIPYDPEHHYKVFSPAASSCHNASGKEAKVCTISPIMGYSTPWRYLDFNALNLFFSPLEFQHLIENYGSIAPDALTVTISEIAVKDVTDKTGGGVQVTDSTTGRLCMLVDHEYKYPYVLGQGQDTLAPELPIWVYFPPQYAYLTVGDVNTQGISGDSKKLASEESAFYVLEHSSFQLLGTGGTATMSYKFPPVPPENLEGCSQHFYEMYNPLYGSRLGVPDTLGGDPKFRSLTHEDHAIQPQNFMPGPLVNSVSTKEGDSSSTGGSGGAHIVMVDAYKPTKGGSGGENLYFQSGGTGGGAGKALTGLSTGTSQNTRISLRPGPVSQPYHHWDTDKYVTGINAISHGQTTYGNAEDKEYQQGVGRFPNEKEQLKRLQGLNMHTYFPNKGTQQYTDQIERPLMVGSVWNRRALHYESQLWSKIPNLDDSFKTQFAALGGWGLHQPPPQIFLKILPQSGPIGGIKSMGITTLVQYAVGIMTVTMTFKLGLRKATGRWNPQPGVYPPHAAGHLPYVLYDPTATDAKQHHRHGFEKPEELWTAKSRVHPLHHHHHHHH |
| **> VP2-L_307_**  MTSVNSAEASTGAGGGGSNPVKSMWSEGATFSANSVTCTFSRQFLIPYDPEHHYKVFSPAASSCHNASGKEAKVCTISPIMGYSTPWRYLDFNALNLFFSPLEFQHLIENYGSIAPDALTVTISEIAVKDVTDKTGGGVQVTDSTTGRLCMLVDHEYKYPYVLGQGQDTLAPELPIWVYFPPQYAYLTVGDVNTQGISGDSKKLASEESAFYVLEHSSFQLLGTGGTATMSYKFPPVPPENLEGCSQHFYEMYNPLYGSRLGVPDTLGGDPKFRSLTHEDHAIQPQNFMPGPLVNSVSTKEGDSSSTGAEHNPVVMVHGIGGASYNFFSIKSYLVGQGWDRNQLYAIDFIDKTGNNRNNGPRLSRFVKDVLDKTGAKKVDIVAHSMGGANTLYYIKNLDGGDKIENVVTIGGANGLVSSRALPGTDPNQKILYTSVYSSADLIVVNSLSRLIGARNVLIHGVGHIGLLTSSQVKGYIKEGLNGGGQNTNGGAGKALTGLSTGTSQNTRISLRPGPVSQPYHHWDTDKYVTGINAISHGQTTYGNAEDKEYQQGVGRFPNEKEQLKRLQGLNMHTYFPNKGTQQYTDQIERPLMVGSVWNRRALHYESQLWSKIPNLDDSFKTQFAALGGWGLHQPPPQIFLKILPQSGPIGGIKSMGITTLVQYAVGIMTVTMTFKLGLRKATGRWNPQPGVYPPHAAGHLPYVLYDPTATDAKQHHRHGFEKPEELWTAKSRVHPLEHHHHHH |
| **>sfGFP-SC**  MRKGEELFTGVVPILVELDGDVNGHKFSVRGEGEGDATNGKLTLKFICTTGKLPVPWPTLVTTLTYGVQCFARYPDHMKQHDFFKSAMPEGYVQERTISFKDDGTYKTRAEVKFEGDTLVNRIELKGIDFKEDGNILGHKLEYNFNSHNVYITADKQKNGIKANFKIRHNVEDGSVQLADHYQQNTPIGDGPVLLPDNHYLSTQSVLSKDPNEKRDHMVLLEFVTAAGITHGMDELYKGGSGGSGGSGGMSYYHHHHHHDYDIPTTENLYFQGAMVDTLSGLSSEQGQSGDMTIEEDSATHIKFSKRDEDGKELAGATMELRDSSGKTISTWISDGQVKDFYLYPGKYTFVETAAPDGYEVATAITFTVNEQGQVTVNGKATKGDAHILEHHHHHH |
